# Smartphone Use, Technology Affordance for Healthcare and Elders' Life Satisfaction

**DOI:** 10.3389/fpubh.2022.861897

**Published:** 2022-04-11

**Authors:** Geling Li, Chenfei Jin, Bin Zhao, Bao Wu

**Affiliations:** ^1^China Institute for Small and Medium Enterprises, Zhejiang University of Technology, Hangzhou, China; ^2^Department of Cooperation and Exchange, Zhejiang University of Technology, Hangzhou, China; ^3^School of Management, Zhejiang University of Technology, Hangzhou, China

**Keywords:** smartphone use, emotional affordance, functional affordance, elders' life satisfaction, living arrangement, selective optimization with compensation

## Abstract

Previous studies have examined how smartphones influence the life satisfaction of the elderly, but the existence of conflicting conclusions suggests the existence of a “black box”. In this study, using a survey from 941 elders, we examine whether smartphone use can improve life satisfaction of the elders by inducing emotional affordance offered by social networking Apps and functional affordance offered by healthcare system Apps. It is found that both emotional affordance and functional affordance acted as intermediating variables between the use of smartphone and elders' life satisfaction. In addition, it is founded that living arrangement with adult children moderates the positive impact of smartphone use on functional affordance, but there was no such moderating effect on emotional affordance. This study offers insights about how digital healthcare innovation will be applied to increase well-being of elders by applying framework of selective optimization with compensation.

## Introduction

During the past decade, information and communication technologies (ICTs) such as smartphones, tablets, and laptops have rapidly penetrated into people's daily life (1–6). ICTs affect the well-being of both individuals and the collective society (7). The aging of the earth's population means that more and more elderly people will be using these new technologies to maintain their health and well-being (4).

Life satisfaction is regarded as the essential form of perceived well-being, and previous studies have focused on the relationship between smartphone use and the life satisfaction of the elderly. Some scholars see a positive effect, and believe that older adults can use smartphones to promote life satisfaction in a variety of positive ways, including expanding their social network (8, 9), obtaining social support (10, 11), reducing loneliness (4, 12), collecting health information or monitoring data (13, 14), and enjoying entertainment (4, 15). Specially, in contrast to call and messenger functions, the Apps embedded in smartphones meet with the diversified needs and demands precisely, which influence the elderly's health and well-being a lot (16). Other researchers see some negative effects, including increasing social isolation, smartphone addiction, and internet deception (17–19). These inconsistent findings may result because older people are seen as passive technology recipients, or because we are ignoring the increasing complexity, interactivity, and feasibility of smartphone-based Apps. Thus, further study is necessary to determine how ICTs (particularly smartphones) can be used to increase the life satisfaction of the elderly.

Although it can be difficult to understand why a specific ICT may provide different utility for different individuals, the actors' role is one of the vital explanations (20, 21). That means the personal subjective perception of technology usage may come into play. The concept of technology affordance—the perceived possibilities (or benefits) that are available from the technology—provides a lens to understand how ICTs can provide for a better life for the elderly (22). However, existing studies typically focus on either (a) technology affordance in terms of people using an ICT (such as a smartphone) to achieve a specific goal (23–25), or (b) product design issues (26–28). Less attention has been paid to understand the mechanism by which technology affordance leads to outcomes (22). Moreover, many studies regard personal technological experience as an important personal variable to explain ICT use, and suggest that various psychological and social reasons explain why older adults adopt new technologies to improve their well-being (29–33). As we know, the major challenge to successful aging is the capability to preserve health or avoiding disease through living autonomously, even if they need much help from other people or technologies (34). In other words, the confirmation of the technology affordance for healthcare including physical and mental assistances in future can offer the elderly more confidence to live well. Due to the booming market of mobile Apps, linked to a plenty of devices, tools and service for physical and mental care, the elderly have more and more opportunities to choose the suitable care styles for them. Thus, technology affordance for healthcare is perceived as a way to see new applications and possibilities in using smartphones to help people age well.

The study reported here uses the framework of selective optimization with compensation theory (SOC) to examine the mediating effect of technology affordance between smartphone use and life satisfaction of the elderly. We also explore the moderating effect of living arrangements between smartphone use and technology affordance for healthcare. This study theoretically and empirically advances our current understanding of gerontechnology usage for aging well from a perspective of subjective activity of the elderly. This study makes three unique contributions to extant literature. First, based on SOC, we identify two pathways by which smartphones affect the life satisfaction of the elderly. In the aging process, the life goals of the elderly are bound to vary compared with those of younger people because of changes in living conditions (e.g., retirement, loss of spouse, and residence in an assisted living facility) and because of a decline of physical and psychological functions (3). In old age, social relationships and the maintenance of functions are regarded as two of the most important life goals, which means staying as happy and healthy as possible. These goals produce two kinds of “selections”—elective selections and loss-based selections (35–37). The differences between such goal-oriented selections lead to different technology searching and application mechanisms for the elderly. The SOC model offers a holistic approach that systematically considers processes rather than specific activities and/or wellbeing outcomes. No previous research has explicitly explored how the present and future usage of various smartphone applications supports SOC processes in later life (38). This study is a first step in filling in that gap.

Second, we investigate the mediating effect of technology affordance for healthcare between smartphone use and elderly life satisfaction. Existing studies separately discuss the application of specific mobile phones in promoting well-being in later life (39, 40). In this study, we break down technology affordance for healthcare into two areas: emotional affordance and functional affordance, which is consistent with elective selections and loss-based selections in the SOC model, and explore the “black box” of SOC with empirical evidence in the context of smart aging in China.

Third, we explore the moderating effect of living arrangements in the context of China. In contrast to the emphasis on individualism in western countries, strong family ties are important in the Chinese filial piety culture (41). We therefore investigate whether living with adult children (or not living with adult children) influences how different smartphone applications may be used to deepen our understanding of social support for technology adoption in old age.

## Theory and Hypotheses

### Smartphone Use, Emotional Affordance, and Elders' Life Satisfaction

Over the past few decades, a significant volume of research has focused on the question of how to age well (35, 36, 42–44). Selective optimization with compensation theory (SOC), originally put forward by Baltes and Baltes (45), is considered as a classic explanation for harnessing people's subjective initiative to live well in later life (37, 38). SOC states that active aging is achieved by using available resources to select targeted life goals, optimizing lifestyles, and adopting alternative approaches to compensate for physical or mental deficiencies (35, 36, 46). Selection refers to the goal-oriented prioritization for activities. It is classified into elective selection (choosing the more important activities and avoiding the less important activities), and loss-based selection (seeking substitutes to compensate for some lack of functionality). Optimization involves acquiring and applying resources or using new technologies for chosen domains, while compensation means finding alternative approaches to meet the essential needs after loss (35, 36, 45, 47).

With the rapid development of science and technology, some researchers are paying attention to how ICTs—which are an important part of an individual's resources—promote aging well during the process of selective optimization with compensation (11, 15, 44, 48). In particular, smartphones can be used for elective selection and optimization for maintaining physical and mental satisfaction, as well as loss-based selection of smart elderly care devices which are functional compensation for living healthy enough. That means that people have more opportunities to select what style of life they want to live in the later stage of life, and they pursue new technologies for both instrumental and social purposes (10). Under the SOC framework, some studies have focused on integrating ICTs into SOC strategies, such as the reduced selection of mobile Apps to meet necessities (15, 49), and increased possibilities for learning to optimize spiritual usage of smartphones (50, 51).

Seniors may crave social support and emotional pleasure first (38). Compared with younger people, the elderly are more likely to regard smartphones as the “buffer” of social isolation through online communication with social ties (3, 52). With more leisure time, older people may need more social activities to maintain social relationships, which reduces depression and loneliness, and increases happiness and satisfaction (3, 12, 13, 42, 53, 54). The current generation of seniors can use smartphone-based Apps to embrace the cyber-culture and enhance their quality of life (55).

Affordance theory (56) originally defined affordance as “possibilities of action” which are provided to individuals by their environment. More recently, the term “technology affordance” has appeared in the IS field; it refers to the opportunities for action provided to a user by ICTs (57, 58). A survey conducted by Barnard et al. (59) found that people with a mean age of 68 believed that technology could be mastered by the elderly, even if they could not do it quite yet, for they had the motivations to pursue proper healthcare. Thus, elderly people perceived that technology affordance for healthcare would give them a greater chance to live well later in life. As suggested by Hutchby (60), Markus and Silver (61), Mesgari and Faraj (62), and Wang et al. (63), technology affordance for smartphones can be classified into two areas. Emotional affordance refers to the emotional or social relationships among actors that are realized by technologies, while functional affordance reflects the possibilities for actions provided to users by the technical features. In summary, the two dimensions of technology affordance correspond to appeals of mental health and physical health. For example, people perceive that an SNS linked to a smartphone affords distinct possibilities for action by satisfying the need for convergence with others within a certain scope (64). They also perceive that robotic technologies, smart speakers, and TV multitasking—which are controlled by smartphones—are an emotional affordance which enriches their spare time and promotes life satisfaction (28, 65). A study conducted by Chan (40), found a positive correlation between social affordance (mobile voice communication) and well-being.

In China, many older migrants, who moved to other communities due to the withdrawal or merger of their original rural villages, and trailing parents, who migrated with adult children in their later years, are more likely to use smartphones to promote social integration and life satisfaction (8, 9, 66). WeChat, Tik Tok, Meitu, and other new smart apps encourage empty-nesters and left-behind elderly to show themselves, express comments, and expand their social networks to improve their ego integrity and life satisfaction (9). Based on the foregoing, we propose the following hypothesis:

*Hypothesis 1: Emotional affordance can mediate the impact of smartphone use on elders' life satisfaction*.

### Smartphone Use, Functional Affordance, and Elders' Life Satisfaction

In recent years, the smartphone has become the most popular ICT in the world, and this has attracted attention from researchers who want to know if it improves people's lives (4, 15, 16, 55, 67, 68). Previous studies differentiated smartphone Apps based on communicative patterns vs. non-communicative patterns. The former refers to channels, activities, or engagement that maintain interactions, enhance connections among people, and generate positive feelings. By contrast, non-communicative/passive patterns emphasize communication tools without communicative purpose; these may cause stress, negative emotions, and compulsive problems in adults (3, 17, 63, 69, 70).

The relationship between smartphone use and life satisfaction may vary with age (3, 4). Some research supports an overwhelmingly positive view of smartphone technology as it affects the elderly (4, 71). Researchers found that older people could use smartphones for purposes like taking advantage of the convenience by e-payment, e-bank, and other e-commerce (4, 72), and staying healthy by managed care applications (73, 74). Other studies that focused on elderly people with disabilities (75), dementia (76), or chronic diseases (77), discussed care technology's potential for alleviating pain or compensating for life inconveniences. Those theoretically derived conclusions are largely confirmed by empirical studies (11).

According to SOC theory, although people decline or lose physical and mental functions with age, they still have the possibility of recovery and development (35, 37, 47, 78). To improve the adaptability while aging, the elderly can allocate and use internal and external resources based on goal-oriented personal development to raise their own functionality to a higher level (36–38, 46). Furthermore, the elderly, to a great degree, tend to compensate for their losses by maintaining an existing state rather than striving for best (37). Assisting technologies are a form of resources that are embedded into social networks. They focus on helping, rather than maximizing the utilization of resources (38). Overall, appropriate compensation strategies result in improved life satisfaction (79).

The functional affordance for healthcare of smartphones for the elderly refers to products or systems integrated by information and communication technologies that improve older people's security (especially fall prevention), daily living and health management. These include human activity monitors, health management systems, smart rehabilitation devices, smart mobility aids, and so on (44, 80, 81). Such functions play an important role of aging in place, and help older adults to maintain independence and autonomy as much as possible and feel more satisfied with life, especially after their physical and mental decline becomes noticeable (15, 64, 82–86). Buck et al. (87) noted the benefits of using gerontechnology, especially the use of help Apps, which improve satisfaction with life for older adults. Lizio et al. (88) compared smart tools for daily telemonitoring at home with health medical equipment near home in the Italian Smart Aging program. They found that most of the elderly subjects preferred smart home programs to improve their quality of life. In other words, the elderly can enhance their independence and autonomy by using intelligent assistive technologies. They can thereby overcome their fear of not being able to take care of themselves and increase their satisfaction of life controllability.

The functional affordance of smartphones has had a strong impact on people's cognitive competence, which raises awareness of ICTs usage and is judged to be the most adequate in reaching the elderly population regarding all issues relating to the incorporation of new technologies in their daily lives (15). den Haan et al. (89) addressed new technology learning and adoption, depending on people's previous technology experience and context. The elderly with good functional affordance of smartphones were more likely to search for, and accept, other gerontechnologies for assistance in compensating for their functional losses (43, 68). A survey by Hernández-Encuentra et al. (15) found that the elderly used ICTs (including smartphones) mainly for leisure (learning, surfing the Internet, travel reservations, etc.), and they would be willing to expand their usage to facilitate daily life-related health and safety issues at home (e.g., telemedicine and tele-assistance, alarm sensors etc.). It seems likely that older adults who do not use smartphones will fall behind in taking advantage of health-related tools; this could lead to decreases in quality of life and jeopardize aspects of their health (71).

We therefore propose the following hypothesis:

*Hypothesis 2: Functional affordance can mediate the impact of smartphone use on elders' satisfaction*.

### The Moderating Effect of Living Arrangement

The details of affordance actualization are contingent on the technology context, which may result in a variety of mechanisms acting as conditions that enable or constrain the actualization of affordance and produce indeterminate outcomes (22). In essence, technology affordance may be a function that is perceived to be provided by existing or future technology, or that is required without knowing whether/when the technology would offer it. That means different affordances may be related to diverse perceptual and attitudinal factors (40), which can be influenced by the people in the system. Specifically, Lawton (90) indicates that living arrangements—an important environmental factor—could act as a resource demanded by individuals which can significantly influence their lifestyle and cognitive functions.

Research on the relationship between living arrangements and cognition or sensibility of the elderly is mixed. Some authors believe that living with adult children is conducive and beneficial to the cognitive function of the elderly (91, 92). Other studies have shown that living with adult children may lead to less free time because of the need to take care of grandchildren and to do household duties; these may have an adverse impact on cognitive function of the elderly (93, 94).

Some studies have found that elderly people living alone may not get enough support from their children, and that may worsen their psychological and cognitive health. Other studies show that older people living alone may have a lower risk of cognitive impairment than with their families, because they must focus on taking care of themselves and completing housework (95). The conclusions vary depending on special context, such as economic and cultural status. For example, Mazzuco et al. (96) compared European countries and found that the relationship between living arrangements and cognitive decline in older people were significantly different in Southern and Northern Europe. Specifically speaking, living with others was more beneficial than living alone in Northern European countries and in some Central European countries, while it was opposite in Southern European countries.

The degree of smartphone usage among older adults also varies by living arrangements. Older people may feel that it is difficult to master multiple applications because of the frequent updating of function integration on smartphones (4, 15). However, learning experiences regarded as self-regulatory processes or life management strategies in SOC theory can overcome the fear of facing new technologies (47). Previous research indicated that older people's learning condition of technology could provide confirmation of their capacity to move on, which meant that there was a positive effect on senior technology affordances (30, 41, 97). In other words, they might voluntarily pursue and operate new kinds of technologies if they were not forced to do so (87, 98). Living with adult children would likely mean more opportunities for enhancing the technology affordance of smartphones *via* the technical supported that is provided by their adult children (e.g., providing hardware, basic information, and technical support) (41).

Changes in economic development and urbanized living arrangements have implications for China's elderly, but there are conflicting patterns. On the one hand, there is an increasing trend of older adults living with their spouse only or living alone rather than living with their children (99). On the other hand, since the family is a vital unit in Chinese culture, many older people have left their own home to live with their adult children. As they take care of their grandchildren, they may come to see that their life could be made happier and more rewarding by the use of a smartphone. Fang et al. (11) suggested that ICT use predicted better well-being among the people 75 old and above. Thus, there may be different priorities for living, depending on whether older people live alone or with their adult children. This may generate different affordances with respect to smartphones (100). We therefore propose the following hypotheses:

*Hypothesis 3a: Living with adult children will positively moderate the relationship between smartphone use and emotional affordance*.

*Hypothesis 3b: Living with adult children will positively moderate the relationship between smartphone use and functional affordance*.

**Figure 1:**
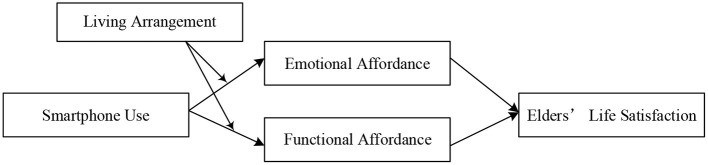
Conception model.

Based on all of the above hypotheses, we propose the model in Figure 1.

## Materials and Methods

### Data and Sample

We collected data from Zhejiang Province, since it is one of the earliest provinces to enter the “aging society,” and since the degree of aging in that province ranks among the top level in China (101). Zhejiang is also one of the most economically developed provinces, is famous for its digital economy, and has promoted the smart care industry to a leading national position (102, 103).

The data are from a survey questionnaire developed by Zhejiang University of technology and Zhijiang laboratory, a research institute established jointly by Zhejiang Provincial Government, Zhejiang University and Alibaba Group. In China, smart elder-care devices are promoted to communities and families due to government pressure. Pilot communities of smart care from 11 cities in Zhejiang Province were randomly selected for this survey. All investigators had received professional training and were required to carry out one-to-one online questionnaire item explanations for the interviewed elderly respondents to prevent misunderstanding.

Table 1 shows the demographic characteristics of individuals in our sample.

**Table 1:** Description of sample.

**Gender**		
Male	470	49.9%
Female	471	50.1%
**Age**		
60–69	443	47.1%
70–79	366	38.9%
≥80	132	14.0%
**Education background**		
Illiteracy	186	19.8%
Primary below	226	24.0%
Primary school	207	22.0%
Middle school or Junior college	289	30.7%
Bachelor or above degree	33	3.5%
**Place of residence**		
Rural areas	412	43.8%
Urban areas	529	56.2%
**Marital status**		
Married	773	82.1%
Unmarried	168	17.9%
Total	941	100%

### Measurements

#### Elders' Life Satisfaction

Referring to previous studies (104, 105), life satisfaction means holding positive emotions rather than negative emotions in general. We use one item which directly evaluates respondent perceptions of the extent to which the elderly have an overall evaluation of life satisfaction. The item is expressed as “All things considered, how satisfied are you with your life as a whole?” This item uses a five-point Likert scale, where 1 = “totally dissatisfied” and 5 = “totally satisfied.”

#### Smartphone Use

Respondents were asked, “Do you use one smartphone at least?” with a response of yes or no. We created a dichotomous variable indicating older adults' smartphone use.

#### Technology Affordance for Healthcare

The value of affordance arises from the perceived content and the process of the interaction of human and products (27, 106). In ICT context, perceiving useful applications is much more important than figuring out the way to use (22, 24, 25, 58). Therefore, we focus on the dimensions of technology affordance in the application and content perspective. Moreover, across a wide variety of domains embedded in smartphones, Apps related with healthcare play a vital role (34). Thus, following Chan (40), Hutchby (60), Markus and Silver (61), Mesgari and Faraj (62), and Wang et al. (63), we divided technology affordance for healthcare into emotional affordance and functional affordance based on apps linking smartphones. Each term contains possible functions or applications or compatible products and service perceived by the participants. Emotional affordance contains three items including company, entertainment and social support, while functional affordance refers to safety, living assistance and health management. Each item is provided through one question: “Which applications or service linked with your smartphone for X can you perceive in future?” And X is for company or entertainment or social support or safety or living assistance or health management. The response for each item contains four to six options, such as spiritual consolation AI, robots, smart speakers and smart chat rooms for company, and fall prevention monitors, night monitors, environment monitors, one-touch call care devices, remote keyless access control and positing bracelet for safety. The participants were asked to choose the possible functions and we counted the total options for each term. Due to positive skew, we transformed the total number of devices/apps perceived possible and useful in future into a 5-point ordinal scale in order to achieve normality.

The reflective multi-item constructs, including emotional affordance and functional affordance, were assessed by analyzing factor loadings, Cronbach's alpha (α), composite reliability (CR), and average variance extracted (AVE). The test results are listed in Table 2. All factor loadings were positive and significant at p < 0.01, supporting unidimensionality of the measures. Cronbach's alphas and composite reliability exceeded the common cut-off levels of 0.7 for all constructs, and the AVE threshold of 0.5 was met in all cases.

**Table 2:** Multi-item measurement information.

**Construct**	**# of items**	**Range of loading**	**Cronbach α**	**CR**	**AVE**
Emotional affordance	3	0.815–0.897	0.824	0.892	0.734
Functional affordance	3	0.749–0.823	0.789	0.8378	0.633

#### Living Arrangement

Since the direct blood relationship is regarded as the most important social relationship in China, and since the adult children play a vital role in elderly technology learning (59, 89, 107), we asked whether participants were living with their adult children as our measure of the variable “living arrangement.” “Yes” or “No” was presented as a 1 or 0 dummy variable, respectively.

#### Control Variables

Participant characteristics might influence their learning capacity and motivation to use a smartphone, and this might relate to life satisfaction. Consistent with the previous literature (3, 32, 41, 66, 100), we controlled demographics including gender, age, educational background, place of residence, and marital status.

### Analytical Strategy

According to Baron and Kenny's suggestion, we tested the mediation and moderation effects in the study by using separate hierarchical multiple regression analyses through STATA 17.0. In all equations, the control variables were filed in before the other independent variables to eliminate their effects from the relationships of principal interest. We finally constructed 11 models.

## Results

The descriptive statistics and correlation matrix are shown in Table 3. The results show that smartphone use is positively associated with emotional affordance and elderly life satisfaction. Life satisfaction of the elderly is positively associated with living arrangement, educational background and marital status, and negatively associated with age and place of residence.

**Table 3:** Descriptive statistics and correlation.

**Variable**	***N***	**Mean**	**SD**	**1**	**2**	**3**	**4**	**5**	**6**	**7**	**8**	**9**	**10**
Life satisfaction of the elderly	941	3.64	0.79	1									
Smartphone use	941	0.51	0.50	0.22^*^	1								
Emotional affordance	941	1.58	0.88	0.08^*^	0.51^*^	1							
Functional affordance	941	1.69	1.16	0.01	0.21^*^	0.46^*^	1						
Living arrangement	941	0.38	0.48	0.08^*^	−0.01	0.01	−0.09^*^	1					
Gender	941	0.50	0.50	0.03	0.13^*^	0.01	0.01	−0.07^*^	1				
Age	941	4.25	0.10	−0.16^*^	−0.41^*^	−0.21^*^	−0.02	−0.14^*^	0.00	1			
Place of residence	941	0.44	0.50	−0.10^*^	−0.20^*^	−0.16^*^	−0.20^*^	0.05	−0.03	0.04	1		
Education background	941	2.74	1.19	0.10^*^	0.41^*^	0.29^*^	0.24^*^	−0.05	0.34^*^	−0.16^*^	−0.27^*^	1	
Marital status	941	0.82	0.38	0.21^*^	0.26^*^	0.11^*^	0.07^*^	−0.11^*^	0.19^*^	−0.32^*^	0.02	0.21^*^	1

* *p < 0.05*.

### The Mediating Effect of Emotional Affordance Between Smartphone Use and Elders' Life Satisfaction

In Table 4, Model 5 only contains the control variables and Model 6 contains results of the main effect of smartphone use on life satisfaction of the elderly. Model 6 shows that there is a positive relationship between smartphone use and life satisfaction of the elderly (coefficient = 0.152, *p* < 0.01) is significant and positive. Models 2, 7, and 8 examine the mediation effect of use of emotional affordance between smartphone use and life satisfaction of the elderly. Model 2 predicts smartphone use has a positive impact on emotional affordance (coefficient = 0.831, *p* < 0.01). Model 7 shows that emotional affordance is significantly and positively related to life satisfaction of the elderly (coefficient = 0.107, *p* < 0.01). Model 8 indicates that smartphone use is not significant, while emotional affordance is significantly and positively associated with life satisfaction of the elderly (coefficient = 0.090, *p* < 0.01). These results support H1, which means that emotional affordance mediates a positive relationship between smartphone use and life satisfaction of the elderly.

**Table 4:** The hierarchical regression results including mediating effect of emotional affordance.

	**Emotional affordance**					**Life satisfaction of the elderly**					
	**Model 1**	**Model 2**	**Model 3**	**Model 4**	**Model 5**	**Model 6**	**Model 7**	**Model 8**	**Model 9**	**Model 10**	**Model 11**
**Control variable**											
Gender	−0.138^**^	−0.155^***^	−0.153^***^	−0.153^***^	−0.025	−0.028	−0.010	−0.014	−0.025	−0.025	−0.012
	(−2.348)	(−2.891)	(−2.860)	(−2.861)	(−0.462)	(−0.520)	(−0.188)	(−0.259)	(−0.468)	(−0.468)	(−0.217)
Age	−1.475^***^	−0.187	−0.102	−0.116	−0.500^*^	−0.264	−0.343	−0.247	−0.127	−0.117	−0.107
	(−5.243)	(−0.688)	(−0.371)	(−0.418)	(−1.952)	(−0.972)	(−1.328)	(−0.913)	(−0.461)	(−0.425)	(−0.390)
Place of residence	−0.164^***^	−0.083	−0.086	−0.089^*^	−0.174^***^	−0.159^***^	−0.156^***^	−0.151^***^	−0.163^***^	−0.161^***^	−0.153^***^
	(−2.864)	(−1.576)	(−1.632)	(−1.697)	(−3.319)	(−3.022)	(−2.989)	(−2.887)	(−3.120)	(−3.067)	(−2.924)
Education background	0.198^***^	0.095^***^	0.094^***^	0.093^***^	0.025	0.006	0.004	−0.002	0.006	0.006	−0.002
	(7.637)	(3.824)	(3.815)	(3.760)	(1.060)	(0.244)	(0.163)	(−0.101)	(0.224)	(0.263)	(−0.066)
Marital status	0.072	−0.019	−0.002	−0.006	0.302^***^	0.285^***^	0.294^***^	0.287^***^	0.313^***^	0.316^***^	0.317^***^
	(0.933)	(−0.271)	(−0.025)	(−0.078)	(4.269)	(4.026)	(4.184)	(4.065)	(4.391)	(4.426)	(4.447)
**Independent variable**											
SU		0.831^***^	0.833^***^	0.833^***^		0.152^***^		0.077	0.156^***^	0.156^***^	0.083
		(14.089)	(14.138)	(14.148)		(2.587)		(1.199)	(2.660)	(2.657)	(1.292)
**Mediator variable**											
EA							0.107^***^	0.090^***^			0.087^***^
							(3.600)	(2.768)			(2.679)
**Moderator variable**											
LA			0.087^*^	0.086^*^					0.140^***^	0.141^***^	0.134^**^
			(1.666)	(1.648)					(2.707)	(2.720)	(2.581)
**Interaction terms**											
SU×LA				0.137						−0.100	−0.112
				(1.345)						(−0.988)	(−1.108)
_cons	7.442^***^	1.873	1.465	1.529	5.564^***^	4.542^***^	4.771^***^	4.373^***^	3.881^***^	3.834^***^	3.701^***^
	(6.073)	(1.585)	(1.215)	(1.268)	(4.985)	(3.846)	(4.219)	(3.712)	(3.229)	(3.187)	(3.084)
*N*	941	941	941	941	941	941	941	941	941	941	941
*R*-square	0.134	0.286	0.288	0.289	0.051	0.057	0.064	0.065	0.065	0.066	0.073

*SU, smartphone use; EA, emotional affordance; LA, living arrangement*.

* *p < 0.10*,

** *p < 0.05*,

*** *p < 0.01*.

### The Mediating Effect of Functional Affordance Between Smartphone Use and Elders' Life Satisfaction

In Table 5, Model 5 only contains the control variables and Model 6 contains results of the main effect of smartphone use on life satisfaction of the elderly. Model 6 shows that there is a positive relationship between smartphone use and life satisfaction of the elderly (coefficient = 0.152, *p* < 0.01) is significant and positive. Models 2, 7, and 8 in Table 5 examine the mediation effect of functional affordance. Model 2 predicts that smartphones will have a positive impact on use of functional affordance (coefficient = 0.391, *p* < 0.01). Model 7 shows that functional affordance is significantly and positively related to the elderly life satisfaction (coefficient = 0.120, *p* < 0.01). Model 8 indicates that smartphone use is positive related to the elderly life satisfaction (coefficient = 0.108, *p* < 0.1), while functional affordance is significantly and positively associated with the elderly life satisfaction (coefficient = 0.114, *p* < 0.01). In all, these results support H2, which means functional affordance mediates the positive relationship between smartphone use and the elderly life satisfaction.

**Table 5:** The hierarchical regression results including mediating effect of functional affordance.

	**Functional affordance**					**Life satisfaction of the elderly**					
	**Model 1**	**Model 2**	**Model 3**	**Model 4**	**Model 5**	**Model 6**	**Model 7**	**Model 8**	**Model 9**	**Model 10**	**Model 11**
**Control variable**
Gender	−0.178^**^	−0.186^**^	−0.186^**^	−0.186^**^	−0.025	−0.028	−0.003	−0.007	−0.025	−0.025	−0.003
	(−2.261)	(−2.383)	(−2.378)	(−2.382)	(−0.462)	(−0.520)	(−0.064)	(−0.124)	(−0.468)	(−0.468)	(−0.065)
Age	0.217	0.822^**^	0.834^**^	0.805^**^	−0.500^*^	−0.264	−0.526^**^	−0.358	−0.127	−0.117	−0.210
	(0.575)	(2.079)	(2.071)	(2.002)	(−1.952)	(−0.972)	(−2.085)	(−1.333)	(−0.461)	(−0.425)	(−0.773)
Place of residence	−0.373^***^	−0.335^***^	−0.335^***^	−0.343^***^	−0.174^***^	−0.159^***^	−0.129^**^	−0.120^**^	−0.163^***^	−0.161^***^	−0.121^**^
	(−4.861)	(−4.380)	(−4.380)	(−4.481)	(−3.319)	(−3.022)	(−2.467)	(−2.301)	(−3.120)	(−3.067)	(−2.316)
Education background	0.230^***^	0.181^***^	0.181^***^	0.178^***^	0.025	0.006	−0.003	−0.015	0.006	0.006	−0.014
	(6.607)	(5.018)	(5.014)	(4.939)	(1.060)	(0.244)	(−0.106)	(−0.592)	(0.224)	(0.263)	(−0.574)
Marital status	0.208^**^	0.165	0.168	0.159	0.302^***^	0.285^***^	0.277^***^	0.266^***^	0.313^***^	0.316^***^	0.298^***^
	(2.004)	(1.600)	(1.605)	(1.528)	(4.269)	(4.026)	(3.966)	(3.806)	(4.391)	(4.426)	(4.221)
**Independent variable**											
SU		0.391^***^	0.391^***^	0.391^***^		0.152^***^		0.108^*^	0.156^***^	0.156^***^	0.111^*^
		(4.546)	(4.546)	(4.559)		(2.587)		(1.834)	(2.660)	(2.657)	(1.890)
**Mediator variable**											
FA							0.120^***^	0.114^***^			0.116^***^
							(5.483)	(5.160)			(5.242)
**Moderator variable**											
LA			0.012	0.010					0.140^***^	0.141^***^	0.140^***^
			(0.159)	(0.131)					(2.707)	(2.720)	(2.736)
**Interaction terms**											
SU×LA				0.298^**^						−0.100	−0.135
				(2.003)						(−0.988)	(−1.343)
_cons	0.315	−2.304	−2.361	−2.220	5.564^***^	4.542^***^	5.526^***^	4.805^***^	3.881^***^	3.834^***^	4.091^***^
	(0.192)	(−1.338)	(−1.342)	(−1.263)	(4.985)	(3.846)	(5.027)	(4.121)	(3.229)	(3.187)	(3.446)
*N*	941	941	941	941	941	941	941	941	941	941	941
*R*–square	0.098	0.118	0.118	0.121	0.051	0.057	0.080	0.084	0.065	0.066	0.093

*SU, smartphone use; FA, functional affordance; LA, living arrangement*.

* *p < 0.10*,

** *p < 0.05*,

*** *p < 0.01*.

### The Moderating Effect of Living Arrangement Between Smartphone Use and Emotional Affordance

Refer back to Table 4, which shows the results of testing H3 (predicting positive moderation of living arrangement between smartphone use and emotional affordance). Models 4 and 11 in Table 4 contain the interaction terms around which the hypotheses were offered. The results of them both suggest that the interactive effect of smartphone use and living arrangement on emotional affordance is not significant. Hence, the results do not support H3a that living with adult children strengthens the positive relationship between smartphone use and emotional affordance.

### The Moderating Effect of Living Arrangement Between Smartphone Use and Functional Affordance

Table 5 also reports the results for H3b which predicts a positive moderation of living arrangement between smartphone use and functional affordance. Models 4 and Model 11 contain the interaction terms around which the hypotheses were offered. Although Model 11 shows that the interaction term is not significant after adding all variables, the results of Model 4 still suggests that the interactive effect of smartphone use and living arrangement on functional affordance is significant and positive (coefficient = 0.298, *p* < 0.05). Thus, H3b is supported.

## Discussions

### Theoretical Contributions

We believe this study makes three contributions to the literature. First, the study distinguishes two different mechanisms to explain how smartphones can promote life satisfaction of the elderly based on elective selection and loss-based selection to improve the subjective well-being of older adults. In terms of social development goals, the emotional affordance of smartphones, which means the possibilities to accept more compatible and prolonged applications or service concerning spiritual pleasure or comfort to improve life satisfaction, old people have more opportunities and choices to accept new technologies by smartphones to enhance social connection and enrich the spirit world. In order to maintain physical and/or mental functions, older people with high functional affordance are willing to learn and accept different types of intelligent care devices to enhance their independence and autonomy; this provides another way to improve their life satisfaction. Previous studies have focused on the qualitative analysis of active aging through technology learning and adoption in the SOC framework, especially discussing specific activities in three processes of SOC (selection, optimization and compensation) separately (77, 83, 108). Since the SOC model offers a holistic approach that systematically considers processes rather than specific activities and/or wellbeing outcomes (38), those two mechanisms in this study have regarded the three processes of SOC as a whole perspective. Thus, starting from the macro level and overall subjective perception, our study contributes to refine SOC theory by quantitative empirical research.

Second, we contribute to the literature by examining the effects of the two different aspects of technology affordance (emotional and functional) in the aging context. Technology affordance exists independently on users and works only if the user perceives and actualizes it (22). So the interactions between technology and users who have unique goals, usage context must be taken into account (57, 58, 61). We therefore classify technology affordance in terms of the perceived possible extensibility of smartphones, rather than in terms of their own attributes (structure, utility, communication, etc.). Our results show that both emotional affordance and functional affordance mediate the relationship between smartphone use and the life satisfaction of the elderly; this provides empirical evidence of technology affordance theory.

Third, we examine the moderating effects of living arrangements on the different types of technology affordance when the elderly use smartphones. These results suggest that smartphone use may broaden functional affordance by strengthening learning capabilities and experiences when the elderly live with adult children. This is consistent with previous studies showing that the internet usage of older adults expanded as a result of encouragement from their children (109, 110). However, we also found that the moderating effect of living arrangements between smartphone use and emotional affordance was not significant. One reason may be that the actualized functions of emotional affordance are less complex than those of functional affordance. For example, irrespective of living arrangements, the elderly always need communication affordance from smartphones (63, 111–113). Moreover, on the one hand, the social needs of the elderly may be diluted by family happiness. On the other hand, dealing with much more housework or taking care of grandchildren encroaches on the time for the elderly to expand their social ties.

### Practical Implications

There are several practical implications of our study. Firstly, the elderly can benefit by accepting the concept of active aging and giving full play to their subjective initiative to use smartphones and related gerontechnologies because more and more mobile applications are linked to intelligent care devices. They would have more chances to obtain the healthcare resource though technology affordance. Meanwhile, this will require that elderly people improve their knowledge about smartphones and their applications. It's never too late to start learning something new, even in the late period of life (59, 114). This improved digital literacy will allow older people to more easily access intelligent care resources. Secondly, the spread of cross-technologies creates opportunities for producers to better serve the elderly. It is necessary to commit to the accurate supply of demand-oriented smart elderly care services for the elderly. Producers should also think about how new technologies can effectively connect with the technologies that the elderly already know. This would create another market for suitable products, and would increase the life satisfaction of the elderly. For example, one-touch call care devices should be targeted at the oldest-old people, while compatible apps should be promoted to younger older people with smartphones. Finally, the government should not only take the lead in launching intelligent care equipment, but also push to create a stronger digital technology learning environment, especially encouraging the family to help the elderly improve their digital literacy. Due to the decreased small number of children in one family and the aggravation of social pension burden, it is imperative to consider appropriate public policies to the combine family support for the elderly and smart aging functions.

## Limitations and Future Research

We recognize that there are several limitations in this study. First, the geographical limitation of data collection may affect the generality of research conclusions. Although Zhejiang Province plays an essential role in digital aging in China, we should not ignore the variations in this issue between regions with different degrees of economic backwardness and aging. Future research is necessary with samples from various regions to increase the generalizability of the results. Due to cultural heterogeneity, future research is also needed to examine the cross-national generalizability of our results. Second, this study focusses only on living arrangements in the context of cross-technology affordance. Future research should analyze digital aging from an integrated perspective, such as taking living arrangements with age together (41). In addition, urbanization and family planning policies have created new social problems for the elderly in China, which may produce more sub-themes based on the social networks of the elderly. Finally, a cross-sectional survey may cause a biased interpretation among variables. Therefore, further studies should attempt to address this issue.

## Data Availability Statement

The raw data supporting the conclusions of this article will be made available by the authors, without undue reservation.

## Ethics Statement

Ethical review and approval were not required for the study on human participants in accordance with the local legislation and institutional requirements. The patients/participants provided their written informed consent to participate in this study.

## Author Contributions

BW contributed to conception, design of the study, and participated the refining of the manuscript. GL wrote the first draft of the manuscript and was responsible for following revision. BZ organized the database and wrote the section of theoretical part. CJ performed the statistical analysis. All authors contributed to manuscript revision, read, and approved the submitted version.

## Funding

This research was funded by Major Project of Zhejiang Provincial Philosophy and Social Sciences Research Planning Project, grant No. 20YSXK02ZD; the Planning Program for Science and Technology of Zhejiang Province of China, grant No. 2021C35024; and Major Fund of National Philosophy and Social Science of China, grant No.17ZDA088.

## Conflict of Interest

The authors declare that the research was conducted in the absence of any commercial or financial relationships that could be construed as a potential conflict of interest.

## Publisher's Note

All claims expressed in this article are solely those of the authors and do not necessarily represent those of their affiliated organizations, or those of the publisher, the editors and the reviewers. Any product that may be evaluated in this article, or claim that may be made by its manufacturer, is not guaranteed or endorsed by the publisher.
